# Effects of Resource Sharing Networks on Community Anti-Drug Coalitions’ Outcomes: A Social Network Analysis

**DOI:** 10.1007/s11121-024-01719-1

**Published:** 2024-08-22

**Authors:** Yvonne Gaddy, Eric C. Jones, Rebecca Wells, Sarah M. Chilenski, Louis D. Brown

**Affiliations:** 1 Department of Health Promotion and Behavioral Sciences, The University of Texas Health Science Center at Houston, School of Public Health, 5130 Gateway Blvd East, El Paso, TX 79905 USA; 2 Department of Epidemiology, Human Genetics and Environmental Services, The University of Texas Health Science Center at Houston, School of Public Health, El Paso, USA; 3 Department of Management, Policy, and Community Health, The University of Texas Health Science Center at Houston, School of Public Health, Houston, USA; 4 Edna Bennett Pierce Prevention Research Center, The Pennsylvania State University, University Park, USA

**Keywords:** Community coalitions, Network analysis, Sectoral diversity, Density, Centralization, Substance use

## Abstract

Substance use-related problems continue to be a national public health crisis despite years of prevention efforts. Community anti-drug coalitions are well positioned to address substance use at local levels. Coalitions often rely on their members to connect to resources they need to address community issues and plan for sustainability over time. Such capacity building occurs through voluntary cooperation among members, making it essential to understand the role network connections play. This study sought to determine whether structural characteristics of coalitions’ resource sharing networks impact members’ perceptions of community improvement and coalition sustainability. Surveys at two timepoints collected data from 68 coalitions in Pennsylvania and Missouri on members’ connections or ties to share information, personnel, money, or other types of collaboration. Analyses examined how coalition-level measurements of sectoral diversity, density, and resource sharing centralization, respectively, were associated with members’ perceptions of community improvement, sustainability planning, and coalition sustainability. Sectoral diversity and centralization were unrelated to study outcomes. Density was also unrelated with perceived community improvement and sustainability planning. However, two facets of cooperative density were positively associated with perceived coalition sustainability: the density of ties to share information and the density of multiple types of collaborative ties. This study suggests that both information and other collaborative ties foster perceived coalition sustainability, although not community improvement.

**Supplementary Information:**

The online version contains supplementary material available at 10.1007/s11121-024-01719-1.

## Introduction

Substance use in the United States has reached alarming levels, causing over 100,000 overdose deaths per year (Friedman et al., 2022) and costs of more than $600 billion annually to families and their communities (Sacks et al., 2015). Community coalitions are a promising approach to address this crisis (Chilenski et al., 2019). Coalitions are well positioned to support a variety of prevention strategies to address an entrenched problem that impacts an entire community. As multisectoral partnerships, coalitions help communities mobilize resources to promote community-driven efforts and enhance resident well-being (Butterfoss et al., 1993). Various public and private entities currently fund community coalitions as a keystone for federal drug prevention efforts (Office of National Drug Control Policy, 2022). However, such support comes through time-limited funding. Coalitions typically have few resources of their own and depend on members for personnel and other resources (Ken-Opurum et al., 2019). Although limited activities can be carried out with volunteers, coalitions’ ability to secure resources affects their capacity to produce community changes and remain sustainable over time (Lardier et al., 2019).

Building coalition capacity puts coalitions’ social capital to work. Social capital is the value derived from interactions among individuals or groups, including trust that can foster mutual commitment. As such, akin to financial or physical capital, social capital is a resource applicable to individual and group goals (Putnam, 1993). Through the social capital generated through ongoing interactions among their members, coalitions have more opportunities to acquire resources necessary to address community issues (Chilenski et al., 2014).

Monetary resources are among the most essential but also most difficult to acquire (Lardier et al., 2019). Evidence shows that a lack of funding negatively impacts coalitions’ goals and sustainability, causing many to be unsuccessful and disband (Lardier et al., 2019). Diversifying the sources and/or types of monetary resources is helpful, such as seeking smaller over larger, more competitive funding grants (Bayne et al., 2012).

Also essential are personnel with skills such as in grant writing, policy advocacy, and program implementation (Bayne et al., 2012). Although a dedicated coordinator is ideal, many coalitions are not able to fund such a position and therefore seek to fulfill these functions through personnel shared among member organizations to cover their respective needs (Community Anti-Drug Coalitions of America, 2018).

Diffusion of information is another key element—from expertise on prevention strategies to knowledge of other resources (Korn et al., 2021). It is not likely, for instance, that the average community member knows of funding opportunities or whether a prevention approach is backed by research (Firesheets et al., 2012). Sharing information can also generate trust and increase cooperation among members and community partners (Myers, 2021).

Sharing of multiple types of resources, such as money, information, and personnel, may be particularly beneficial (Liu et al., 2019). Such “multiplexity” gives coalitions greater access to these resources, as interactions can build on partnerships where trust already exists (Bayne et al., 2012).

Who and what organizations constitute a coalition’s membership plays an important role in coalitions’ capacity. Coalitions not only rely on their members to carry out tasks but also to acquire resources and implement activities in the community (Zakocs & Edwards, 2006). Representation from diverse sectors helps build widespread support from a variety of perspectives and enables coalitions to draw on a broader range of resources (Brown et al., 2010).

Member relationships, or connections, are the basis for coalition networks. Networks help create trust among members and are the means through which collaboration typically occurs within the coalition and with the broader community (Kumar & Sinha, 2021). Ideally, coalitions should continually work at building and sustaining their networks (Provan et al., 2005). Adequate network structure enables coalitions to acquire resources and implement change which, in turn, enhances members’ perceptions of coalition effectiveness and sustainability over time. If a coalition is perceived as effective, its members are more likely to continue investing their time and cooperation (Wells et al., 2009).

### Social Network Analysis

Social network analysis may be useful for coalitions to understand how patterns of interactions yield social capital, and how these resources can be deployed to improve coalition and community capacity (Provan et al., 2005). Network analysis provides a unique lens to assess the composition and collaborative structure of resource sharing networks to identify interaction patterns among coalition members and community partners (Borgatti et al., 2018). We examine the resource sharing networks of coalition members who represent diverse community sectors connected by a cooperative relationship or tie with other sectors to share resources—monetary, personnel, information, or multiplexity.

While research has applied some network analysis to examine collaborative connections within coalition networks (Brown et al., 2017; Heeren et al., 2022; Manning et al., 2014; Valente et al., 2007), information is lacking on the effects resource sharing networks have not only on members’ perceptions of community improvement and coalition sustainability, but also on planning for coalition sustainability. To fill this gap, this study assessed network composition—or who is in the network—through sectoral diversity or what kinds of entities are in the network; and assessed network collaborative structure through density of ties and centralization of those ties within a few sectors.

### Conceptual Framework

The current study is informed by LaFasto and Larson’s (2001) model for group effectiveness. While this model is based on traditional teamwork within organizations, it is appropriate given its comprehensiveness and focus on social dynamics. One key dynamic within the model is the importance of bringing the right people or, in the case of coalitions, the right community sectors together to work toward common goals. Given coalitions’ reliance on their members, diversifying memberships across community sectors, or sectoral diversity, enhances this dynamic of bringing the right sectors together to collaborate in coalitions’ resource sharing networks (Chaisson et al., 2022).

Another key dynamic for group effectiveness is a network structure that is conducive to and encourages positive group interactions to increase the likelihood of achieving goals. For coalitions, the density and centralization of members’ connections or ties help form the structure of their networks. Density refers to the proportion of all possible ties within the network, while centralization measures the extent ties are focused around a few members or more broadly across the network (Borgatti et al., 2018). A network that is conducive to positive group connections to acquire the necessary resources typically depends on having adequate density without too much centralization (Korn et al., 2021; Valente et al., 2015). Based on this model, this study used a social network approach to assess the effects network structure has on perceived community improvement, perceived coalition sustainability, and planning for such sustainability.

### Study Hypotheses

Sectoral diversity distributes coalition memberships across relevant community sectors, increasing the possibilities from which coalitions can draw perspectives and resources (Brown et al., 2017). By including diverse sectors, coalitions have access to different types of resources each sector can provide (i.e., information, personnel, monetary, or other types) and can leverage more efficient use of these resources (Korn et al., 2021). Identifying disconnected sectors may help coalitions devise plans to better engage community partners, thereby strengthening network composition. Sectoral diversity may also create difficulties collaborating due to opposing perspectives (Hearld et al., 2019), thus the potential for reduced communication and cohesion among the group (Brown et al., 2017). However, if members perceive the coalition to be effective, these differences can be managed, with the rationale for collaboration prevailing (Provan et al., 2003). Despite mixed findings but based on the potential of multiple sectors to actualize communities’ diverse resources, we hypothesized that:

- Hypothesis 1: Sectoral diversity of resource sharing ties—information, personnel, monetary, multiplexity—at time 1 will be associated with higher perceptions of community improvement, sustainability planning, and coalition sustainability at time 2.

Density describes the level of connectedness within a network (Hanneman & Riddle, 2005). Dense networks have more pathways to share resources and the potential for ties to subnetworks compared to sparse networks with fewer pathways (Fujimoto et al., 2009). Whether or not dense networks lead to better outcomes is another question. That is, greater network density may lead to suboptimal outcomes due to redundant connections within the group and fewer connections to new resources and opportunities external to the coalition (Valente et al., 2007). However, greater density may also play an important role in creating higher levels of trust, increasing the probability of more sustainable connections among members from different sectors (Heeren et al., 2022). Nevertheless, overly dense networks may give rise to conflicts when opposing views are entrenched (Feinberg et al., 2005), although most coalitions have relatively low density. Finally, managing and coordinating cooperation may be time-consuming as the number of ties grows, thereby increasing the chances of ignoring important connections when they occur (Provan et al., 2005). Despite mixed evidence, this study posits:

- Hypothesis 2: Density of each type of resource sharing tie—information, personnel, monetary, or multiplexity—at time 1 will be associated with higher perceptions of community improvement, coalition sustainability, and sustainability planning at time 2.

Centralization captures the extent to which resource sharing ties are concentrated around one or a few members or more evenly dispersed across the network (Freeman, 1978). Centralized networks typically provide hubs that facilitate collaboration through numerous connections (Valente et al., 2007). Yet, overly centralized networks can concentrate control and reduce shared decision-making, potentially resulting in less commitment among non-central members and fewer connections to community partners (Fujimoto et al., 2009; Valente et al., 2007). The hierarchical approach of centralized networks may also inhibit interactions. A decentralized network may be more functional, distributing power and influence across all members (Feinberg et al., 2005). As a result, members feel their participation is meaningful and continue to invest themselves in coalition activities. Based on the emphasis of LaFasto and Larson’s model that group effectiveness depends on an appropriate network structure and on existing evidence, we hypothesized that:

- Hypothesis 3: Centralization of each type of resource sharing tie—information, personnel, monetary, or multiplexity—at time 1 will be associated with lower perceptions of community improvement, sustainability planning, and coalition sustainability at time 2.

## Methods

### Study Design

This study analyzed data from the Coalition Check-Up (Brown et al., 2021), an ongoing randomized trial among anti-drug coalitions that focuses on increasing member engagement, helping communities invest their resources wisely, and promoting healthy youth development through optimal implementation of evidence-based prevention to reduce youth substance use.

### Study Participants

The population targeted for participation in the Coalition Check-Up were 68 community anti-drug coalitions across Pennsylvania and Missouri. These coalitions, ranging from 1 to 37 years of age, operate in areas that are rural or mostly rural (49%) and urban or mostly urban (51%). Coalitions also varied by the community prevention model they follow. The most commonly employed model was Communities That Care (CTC) (41%); other models included Drug-Free Communities (DFC) (34%), PROmoting School-community-university Partnerships to Enhance Resilience (PROSPER) (7%), Strategic Prevention Framework (5%), or no model at all (24%).

Coalitions were recruited by the Evidence-based Prevention and Support group (EPIS) in Penn State University’s Prevention Research Center through email communications, coalition meetings, phone calls, web meetings, and in-person visits. To be eligible, coalitions had to be operational for at least one year, have a designated coordinator, hold meetings at least quarterly with multiple community sectors in attendance, and currently support implementation of drug prevention activities or secured funding to do so. Coalitions also had to be willing to be randomized to the intervention, although the current analyses include comparison coalitions. Coalitions made the final decision to participate and provided rosters with active members, i.e., members who had attended at least two meetings in the previous 12 months.

### Data Collection

In Pennsylvania, time 1 data was collected from October to December 2020 and in Missouri from April to June 2021. Time 2 data was collected 12 months later in Pennsylvania from October to November 2021 and in Missouri in April and May 2022.

Sixty-eight coalitions were initially recruited across Pennsylvania and Missouri. Of these, 63 coalitions had data at both timepoints, resulting in a final sample of 63 coalitions. Coalition members received a unique link to an online survey via email. A total of 1081 out of 1738 members consented to participate at time 1, for a response rate of 62.2%. A total of 945 out of 1695 members consented to participate at time 2, for a response rate of 55.8%. Member responses were aggregated to the coalition level given that the focus was on coalition level outcomes. All study procedures were approved by The Pennsylvania State University Institutional Review Board.

### Measures

Study outcomes were based on previously validated scales (Brown et al., 2012). The outcome of perceived community improvement used a set of eight items to measure perceptions over the previous 12 months (Wells et al., 2009). Respondents were asked “…how has your community changed over the past 12 months due to your coalition’s efforts?” (*α* = 0.90) in each of eight areas—funding level for youth programming, local policies that impact substance use, environmental conditions that impact substance use, use of evidence-based youth programming, quality of local services and programs, evaluation of local services and programs, level of youth substance use, and residents’ well-being. Using a 5-point Likert scale, response options ranged from 1 (much worse) to 5 (much better).

Sustainability planning was assessed through four items: has a concrete plan been developed to continue offering programs, has the plan been implemented, have potential funding sources been explored for existing programs, and has a plan been developed to continue engaging volunteers and strengthen the membership (*α* = 0.87). Response options ranged from 1 (no) to 4 (a lot).

Lastly, the outcome of perceived coalition sustainability was based on one item—“How likely do you think it is that the coalition will continue for the next 3 years?”—with responses ranging from 1 (highly unlikely) to 4 (highly likely). For each of the above outcomes, the mean across all coalition members was calculated to create continuous variables for each coalition.

Structural characteristics of coalitions’ resource sharing networks as independent variables were sectoral diversity of each network, network density, and network degree centralization. We define sectoral diversity as the extent to which a coalition has representation from various sectors of the community. Each respondent indicated which of 23 community sectors within the study (e.g., education, healthcare, community resident) best described their representation as a member of the coalition. We then used an entropy index to compute our measure of sectoral diversity, which increases as sector representation becomes more evenly distributed across a larger number of sectors (Ramaciotti Morales, 2021), as used in previous coalition studies (Brown et al., 2017):

$$Entropy=\sum_{i=1}^{k}\left({p}_{i}\right)\left(\frac{1}{{p}_{i}}\right)$$

where *i* = 1 to *k* sectors and *p*_*i*_ is the proportion of each coalition’s members in the *i*th sector. The final entropy index for each coalition results from the proportion of members from each sector and the natural log of the inverse thereof to ultimately indicate sector distribution among members. The minimum index value equals 0 if all members belong to the same sector and increases as the number of sectors and the membership distribution across sectors increases, with a maximum entropy equal to the natural log of the number of sectors. This study included 23 community sectors as shown in Table 1, making 3.1 the maximum possible sectoral diversity value.

**Table 1:** Coalitions’ descriptive statistics at time 1 (*n* = 63)

Variable	Mean or %	Standard deviation	Minimum	Maximum	
Coalition age (years)	11	8.5	1	37	
Coalition size (number of survey participants)	16	9.6	2	53	
Poverty level in the region	12%	.06	.02	.33	
State—Pennsylvania or Missouri	86% PA	NA			
Number of sector representatives					
Elementary, middle, high schools, school districts	2.8	2.9	0	13	
Substance use-related organizations	1.9	2.1	0	9	
Mental/behavioral health services	1.6	2.0	0	9	
Other (including national guard or other military)	1.4	1.4	0	5	
Healthcare, hospitals, other health organizations	1.0	1.4	0	6	
Law enforcement	0.7	1.0	0	4	
Youth serving organizations	0.6	0.9	0	3	
Child welfare	0.6	1.1	0	6	
Other education (e.g., universities, technical colleges)	0.6	1.2	0	7	
Religious/faith organizations	0.5	0.8	0	4	
Other local government	0.5	1.1	0	7	
Unaffiliated parent or community resident	0.5	1.1	0	6	
Local government; parks and recreation	0.5	0.7	0	3	
Child/family advocacy	0.4	0.8	0	4	
Business	0.4	0.7	0	3	
Judicial System	0.4	1.0	0	6	
Civic or volunteer groups	0.2	0.5	0	2	
Other federal or state government agencies	0.2	0.5	0	2	
Cooperative Extension	0.2	0.6	0	3	
Public health departments (local or state)	0.1	0.5	0	3	
Media	0.1	0.3	0	2	
Youth (represent individuals under age 18)	0.1	0.3	0	2	
Firefighters or paramedics	0.0	0.3	0	2	
Sectoral diversity (entropy)	1.7	0.5	0.6	2.4	
Density					
Information	0.6	0.1	0.3	0.9	
Personnel	0.2	0.1	0.0	0.5	
Monetary resources	0.1	0.1	0.0	0.3	
Multiplexity	1.2	0.3	0.5	2.1	
Degree centralization					
Information	0.7	0.1	0.4	0.9	
Personnel	0.5	0.2	0.1	0.9	
Monetary resources	0.3	0.2	0.0	1.0	
Multiplexity	0.4	0.1	0.2	0.7	
Perceived community improvement	3.4	0.3	2.7	4.1	
Perceived coalition sustainability	3.5	0.4	2.4	4.0	
Sustainability planning	1.9	0.4	0.8		2.6

*PA*, Pennsylvania, *NA* not applicable

Density and degree centralization measured the collaborative structure of coalitions’ resource sharing network. Respondents indicated the type of collaboration they or their workplace had with each of 23 sectors on youth substance abuse prevention regardless of whether the relationship was coalition related. Response options were: *share information outside coalition meetings*, *share monetary resources*, *share personnel*, and *other cooperation* [adapted from (Chilenski et al., 2014)]. Each option was considered a different type of tie. When one or more respondents from a sector named another sector, the sectors were joined by a tie (i.e., presence (1) or absence (0) of a tie). This involved changing the bipartite or two-mode network (members-sectors) into a unipartite or one-mode network (sector-sector). Thus, a binary adjacency matrix of ties among the 23 sectors to share each type of resource was constructed to then quantify density and degree centralization using UCINET software version 6.766 (Borgatti et al., 2002).

Network density is calculated as each coalition’s number of member-reported ties to share each type of resource—personnel, monetary, information, and multiplexity—divided by the maximum number of ties possible using the formula (Wasserman & Faust, 1994):

$$Density=t/n(n-1)$$

where *t* is the number of actual ties and *n* is network size (number of coalition members). Multiplexity was calculated taking the coalition’s average number of unique *types* of collaboration with each other member (Manning et al., 2014) among the four types of ties shared: monetary resources, personnel, information, and other cooperation. Multiplexity scores ranged from 0 (no collaboration) to 4 for each type of reported tie.

Degree centralization captures the extent to which the pattern of cooperative ties is concentrated on the network’s most central members (Freeman, 1978). Degree centralization ranges from 0 to 1 with higher numbers indicating a more centralized network and is based on degree centrality, which measures the extent individual members are connected through direct ties. Degree centralization was calculated using the formula (Freeman, 1978):

$$Degree\ centralization=\sum (Cmax-Ci)/(n2-3n+2)$$

where *C* was member-level degree centrality and* n* was network size. Degree centralization is the ratio of the sum of differences between the degree centrality scores of the most central member (*C*_max_) and that of all other members (*C*_*i*_), divided by the maximum possible sum of differences (Fujimoto et al., 2009).

Coalition age in years, poverty level of coalitions’ service area, coalition size in number of respondents, state (Missouri or Pennsylvania), and experimental condition were included as covariates to reduce potential confounding. As coalitions age, they go through a series of stages and likely become more effective if they remain active. Age was therefore included as a covariate given that perceived community improvement and coalition sustainability may differ depending on coalitions’ stage of maturity (Brown et al., 2010). The poverty level in coalitions’ service area may affect the number of well-functioning local institutions with which to partner, thus helping or hindering coalition efforts (Greenberg et al., 2007). Poverty level was determined using 2020 Census data (US Census Bureau, 2022). Weighted averages were used for coalitions whose service area extends across more than one region. Coalition size should also be accounted for when comparing networks as centralization is partly a function of the size of the network it is calculated on (Freeman, 1978). A comparative assessment was conducted to determine whether coalition rosters would be a more accurate and reliable measure of coalition size as opposed to the number of survey respondents. The associations were similar and did not change the results. Thus, we based coalition size on survey respondents given the likelihood that respondents are more active in a coalition and perhaps a more reliable source of information. Furthermore, coalition rosters were not always current, as we observed during data collection. Lastly, data were taken from early stages of coalitions’ participation in the Coalition Check-Up study—time 1 data collected before randomization and time 2 one year into the intervention. We theorized that the intervention had not yet affected the levels of association between variables, consequently including experimental condition as a covariate.

### Data Analysis

We used SAS™ Enterprise Edition version 3.81 (SAS Institute Inc., 2022) to conduct all analyses once network measures of density and degree centralization were calculated in UCINET software version 6.766 (Borgatti et al., 2002). Analyses were conducted at the coalition level given the focus of coalition level outcomes. We used all available data through a measure of central tendency (i.e., the mean) of individual responses to survey items. The dataset was first examined for outliers and missing data. Once aggregated to the coalition level, however, missing data was not a problem although one coalition did not have personnel-sharing ties. Given the small size of this coalition and the poverty level, the lack of these ties was not considered unusual and remained missing. To determine if specific portions of the data behaved differently, data were examined in scatterplots with vertical lines added to denote quintiles created among the outcomes to see if associations varied by quintile. No clear visual patterns were observed.

Multiple linear regression quantified the effect time 1 measures of sectoral diversity, density, and degree centralization of personnel, information, monetary resources, and multiplexity ties had on coalition level outcomes at time 2, i.e., 12 months later. These outcomes were perceived community improvement, sustainability planning, and perceived coalition sustainability. Time 1 measures of outcomes were also included as independent variables in each regression model to predict change over time.

Sectoral diversity, density, and degree centralization were tested in separate models. The independent variable of interest was entered into the model first. Covariates were then added one by one. If the covariate changed the hypothesis testing estimate by more than 10%, it was kept in the model. We tested all regression assumptions following procedures outlined by Chen et al. (2003) and found that all models were consistent with assumptions.

## Results

Table 1 presents the descriptive statistics for study measures. The mean coalition age was 11 years and an average of 12% of the local population in coalitions’ service area had incomes below the federal poverty threshold compared to the national average of 11.4% (US Census Bureau, 2022). The average coalition size was 16 members. The most commonly represented sectors were schools—elementary, middle, high schools, school districts (mean, 2.8 per coalition)—and substance use-related organizations (mean, 1.9 per coalition).

Measures of study outcomes at time 1 varied from a mean of 3.4 (out of 5) for perceived community improvement, 3.5 (out of 4) for perceived coalition sustainability, and 1.9 (out of 4) for sustainability planning. Density, which ranges from 0 to 1 for single measures and 0 to 4 for multiplexity, was highest for information sharing (mean = 0.6 ties per coalition) and multiplexity ties to share resources (mean = 1.2 ties per coalition). Degree centralization, ranging from 0 to 1, was highest among information sharing ties (mean = 0.7). Density and degree centralization of monetary resource sharing ties were lowest (mean = 0.1 and 0.3, respectively).

Table 2 reports the multiple variable regression results that tested our hypotheses. The first hypothesis that sectoral diversity within coalitions’ resource sharing networks would be associated with significant increases in study outcomes at time 2 was not supported. In fact, the diversity index was negatively associated with all study outcomes at time 2 although the association was not significant. Our second hypothesis was partially supported, as density of information ties (B = 0.75, *β* = 0.30, *p* = 0.002) and density of multiplexity (B = 0.24, *β* = 0.23, *p* = 0.02) were positively associated with perceived coalition sustainability. That is, for a one standard deviation increase in density of ties to share information, the model predicted a 0.30 standard deviation increase in perceived coalition sustainability over time, holding coalition state and poverty level constant. Likewise, a one standard deviation increase in density of multiple types of resource sharing ties predicted a 0.23 standard deviation increase in coalition sustainability, holding coalition size constant. Our third hypothesis that degree centralization would be associated with significant reductions in study outcomes was not supported by any type of resource sharing tie. Four of the 12 models with degree centralization were negatively associated with coalition outcomes, although these coefficients were not statistically significant and therefore did not meet the threshold to support our hypothesis.

**Table 2:** Multiple regression models of time 2 outcomes with time 1 predictors (*n* = 63)

Predictor (time 1)	Outcome (time 2)								
	Perceived community improvement			Perceived coalition sustainability			Sustainability planning		
	B	95% CI	*β*	B	95% CI	*β*	B	95% CI	*β*
Sectoral diversity	− 0.06	− 0.25 to 0.14	− 0.10	− 0.08	− 0.29 to 0.13	− 0.10	− 0.10	− 0.34 to 0.14	− 0.11
Density									
Monetary	0.26	− 0.49 to 1.02	0.08	− 0.31	− 1.16 to 0.54	− 0.07	0.24	− 0.67 to 1.16	0.05
Personnel	0.10	− 0.58 to 0.79	0.03	0.03	− 0.74 to 0.80	0.01	0.42	− 0.39 to 1.23	0.09
Information	0.17	− 0.29 to 0.62	0.08	0.75^*^	0.30 to 1.21	0.30	0.16	− 0.40 to 0.72	0.05
Multiplexity	0.07	− 0.12 to 0.26	0.09	0.24^*^	0.04 to 0.44	0.23	0.04	− 0.18 to 0.26	0.03
Degree centralization									
Monetary	0.20	− 0.13 to 0.53	0.14	0.02	− 0.37 to 0.40	0.01	0.15	− 0.25 to 0.55	0.07
Personnel	0.03	− 0.27 to 0.33	0.02	− 0.14	− 0.48 to 0.19	− 0.09	0.20	− 0.14 to 0.53	0.10
Information	0.18	− 0.36 to 0.72	0.08	0.55	− 0.06 to 1.16	0.19	− 0.10	− 0.79 to 0.58	− 0.03
Multiplexity	0.30	− 0.20 to 0.80	0.13	− 0.08	− 0.63 to 0.47	− 0.03	− 0.05	− 0.67 to 0.57	− 0.01

^*^p < .05

Time 1 measures of perceived community improvement, perceived coalition sustainability, and sustainability planning, included in the regression models to measure change over time, showed stability in predicting the respective outcome at time 2, ranging from B = 0.35 to 0.76 (*β* = 0.37 to 0.74). The relations between covariates and outcomes varied. Coalition age, size, state, and experimental condition were typically not significantly related to outcomes; however, community poverty was related. For example, in the sectoral diversity models, poverty was negatively associated with perceived community improvement (B =  − 1.36, *β* =  − 0.28, *p* = 0.02), coalition sustainability (B =  − 1.48, *β* =  − 0.24, *p* = 0.02), and sustainability planning (B =  − 1.26, *β* =  − 0.17, *p* = 0.07). Correlations among major variables are available in Online Resource 1.

## Discussion

This study evaluated the effects structural characteristics of coalitions’ resource sharing networks have on members’ perceptions of community improvement and coalition sustainability. We examined associations between sectoral diversity, density, and degree centralization of resource sharing ties with coalition outcomes in a sample of 63 anti-drug coalitions in Pennsylvania and Missouri.

The results failed to support the first hypothesis that sectoral diversity within coalitions’ resource sharing networks would be associated with significant increases in perceived community improvement, perceived coalition sustainability, or sustainability planning. These null results may reflect the challenges that diverse member perspectives and approaches bring (Brown et al., 2017; Feinberg et al., 2008). More sectoral diversity, in the absence of actual connections to share resources, may inhibit members’ perceptions of coalition outcomes (Valente et al., 2007). Findings may also suggest differences in how coalitions’ connections facilitate their capacity to create community change. That is, one coalition’s members may be highly active but lack sectoral diversity, while another may have diversity but limited member engagement (Korn et al., 2021). Such differences may reflect coalitions’ varying developmental stages as well as growth over time; hence, it may be possible that the 12-month timeframe in our study was not long enough (Brown et al., 2017; Feinberg et al., 2008).

Findings on the hypothesized benefits of the density of ties to share information and multiple types of collaboration with perceived coalition sustainability may reflect higher levels of trust and potentially more sustainable connections (Heeren et al., 2022). Based on LaFasto and Larson’s model (2001), the combination of positive interactions and strong relationships might lead to more cooperation among members to achieve coalition goals and sustainability and, in turn, more connections to share resources. However, once sufficient density is reached to effectively share resources, more ties may become difficult to maintain (Provan et al., 2005). Furthermore, information sharing alone may not translate into sustainability, thus emphasizing multiplexity might help promote the coordinated mobilization of resources (Liu et al., 2019). Multiplexity benefits coalitions given that the loss of one type of tie, such as personnel sharing, would likely impact the coalition less because other types of ties also exist, as does trust between members (Liu et al., 2019). To that regard, the low mean density of ties to share both monetary resources and personnel among coalitions in our study suggest a need to establish connections across more sectors. Other factors may have also been influential. For instance, data collection was carried out during the COVID-19 pandemic when monetary and personnel resources were scarce, prevention programs shifted to virtual sessions or no sessions at all, in-person events were canceled, and coalition goals likely shifted due to programmatic interruptions (Christian et al., 2022).

The absence of hypothesized centralization benefits found in the current study may indicate that the efficiency benefits of centralization counterbalance the inclusivity benefits of decentralization. For example, if implementation is focused on a specific sector such as schools, having centralized resource sharing within that sector may have some practical benefits. We found no previous studies measuring these same associations, although past findings on the impact advice network centralization had on adopting evidence-based practices were mixed. One study found centralization had no significant effect (Valente et al., 2007), while another found that decreased concentration of influential leaders (i.e., decentralization) helped in the diffusion of coalition programs (Fujimoto et al., 2009).

A noteworthy finding was the relationship between poverty in a community and each of the coalition outcomes. Prior research suggests that higher levels of community poverty are related to reduced funding for community services, creating more conflict among organizations to acquire what little is available (Greenberg et al., 2007). Funding constraints negatively affect coalitions’ capacity to create community change and, as a result, impact members’ perceptions of coalition effectiveness and create higher levels of burnout (Greenberg et al., 2007). Also worth noting is the lack of effects coalition age, size, and state had on study outcomes. These findings suggest generality not only across coalitions of varying ages and sizes but also across states, and thus potentially to more areas of the U.S.

### Strengths and Limitations

Several strengths make this study a valuable contribution to the literature. One key strength is the large sample of coalitions from Pennsylvania and Missouri. These coalitions are diverse in their application of different coalition models and have varying sizes and ages, as well as rural and urban locations. This diversity helps to increase the generalizability of the study findings. Further, this study used well-developed outcome measures implemented in prior research (Brown et al., 2012). Lastly, data was collected over two timepoints, making it possible to analyze changes over time and strengthening causal inference.

Important limitations also exist. Active coalition members may be more likely to participate in our study versus those not as active, which can limit our understanding of network dynamics and relationships among less active members. It was also impossible to know if resource sharing ties provided by survey respondents differ from ties among members who did not participate in the study, which may skew the results. Additionally, data collection occurred during the COVID-19 pandemic when most coalitions had online meetings and activities and perhaps reduced levels of activities, potentially impacting their effectiveness in prevention efforts (Imm et al., 2020). The pandemic possibly had a negative impact on coalitions’ presence in the community and may have been reflected in members’ perceptions of coalitions’ effectiveness and ability to survive through the pandemic and afterward (Imm et al., 2020). On the other hand, virtual meetings held during the pandemic may have increased meeting participation among members who could not otherwise attend. It should be noted that the sample size of 63 coalitions, while large for this type of study, provided limited power. Our findings also do not include the impact on community-level indicators, such as past 30-day use of alcohol or opioid use in coalition’s catchment area, which we expect to explore in future analyses. Nor do our findings address coalitions’ flexibility to handle community changes over time. Future research might focus on the relationship between coalitions’ resource sharing and community outcomes reflecting coalition capacity to flexibly meet changing community needs. Finally, this study used self-reported data which can lead to biases from memory recall.

## Conclusions

Community coalitions often rely on their members for resources needed to address community issues and plan for sustainability over time. Similar to other studies (Brown et al., 2017), however, our findings indicate that sectoral diversity may be challenging for success, particularly if different sectors are present but not actively sharing resources. Nonetheless, many forms of cooperative connections between sectors, as well as multiple types of cooperative connections, should be encouraged given the potential for those connections to support coalition sustainability. Our findings may also illustrate the limitations of centralization. That is, some networks may benefit from the efficiency of centralization, while others may benefit from the inclusivity of decentralization. Future research should explore other influential characteristics of network structures, potentially using different social network measures.

## Supplementary Information

Below is the link to the electronic supplementary material.

Supplementary file1 (PDF 214 KB)

## Data Availability

The data that support the findings of this study are available from the corresponding author upon reasonable request.
