# Supplementary material for: Effects of Resource Sharing Networks on Community Anti-Drug Coalitions’ Outcomes: A Social Network Analysis
Source: Prev Sci. 2024 Aug 22;25(7):1029–39. doi: 10.1007/s11121-024-01719-1 (PMC11519087; doi:10.1007/s11121-024-01719-1)
Supplement: Supplementary file 1 — Supplementary file1 (PDF 214 KB) [file 11121_2024_1719_MOESM1_ESM.pdf]

Effects of Resource Sharing Networks on Community Anti-Drug Coalitions' Outcomes: A Social Network Analysis

Prevention Science

M. Yvonne Gaddy[1], Eric C. Jones[2], Rebecca Wells[3], Sarah M. Chilenski[4], and Louis D. Brown[1]

[1] Department of Health Promotion and Behavioral Sciences, The University of Texas Health Science Center at Houston, School of Public Health

[2] Department of Epidemiology, Human Genetics and Environmental Services, The University of Texas Health Science Center at Houston, School of Public Health

[3] Department of Management, Policy, and Community Health, The University of Texas Health Science Center at Houston, School of Public Health

[4] Edna Bennett Pierce Prevention Research Center, The Pennsylvania State University

Corresponding author:
Yvonne Gaddy, DrPH
mailto:mary.y.gaddy@uth.tmc.edu

Zero-order correlations among study measures

	M	*SD*	1.	2.	3.	4.	5.	6.	7.	8.	9.	10.	11.	12.	13.	14.
Dependent Variables (Time 2)																
1. Perc. community improvement [a]	3.4	0.3	—													
2. Perc. coalition sustainability	3.5	0.4	.38**	—												
3. Sustainability planning [a]	1.9	0.4	.53***	.65***	—											
Independent Variables (Time 1)																
4. Sectoral diversity (entropy)	1.7	0.5	.16	.06	.17	—										
Density:																
5. Information	0.6	0.1	.10	.32**	-.02	-.01	—									
6. Personnel	0.2	0.1	.08	.13	.13	-.02	.38**	—								
7. Monetary	0.1	0.1	.18	.10	.09	-.05	.40***	.60***	—							
8. Multiplexity	1.2	0.3	.17	.29*	.06	-.01	.81***	.73***	.70***	—						
Degree centralization:																
9. Information	0.7	0.1	-.05	.09	-.03	-.54***	.18	.06	.10	.17	—					
10. Personnel	0.5	0.2	.15	.09	.22	.28*	.18	.70***	.35**	.43***	.01	—				
11. Monetary	0.3	0.2	.28*	.23	.23	.12	.29*	.49***	.80***	.54***	.05	.44***	—			
12. Multiplexity	0.4	0.1	.10	.00	-.05	-.22	.31**	.57***	.44***	.53***	.37**	.50***	.48***			
13. Perc. community improvement [a]	3.4	0.3	.37**	.37**	.60***	.34**	-.01	.11	.22	.14	-.13	.20	.27*	.02	—	
14. Perc. coalition sustainability	3.5	0.4	.29*	.62***	.51***	.14	.03	.21	.24	.15	.02	.22	.30*	.06	.54***	—
15. Sustainability planning [a]	1.9	0.4	.36**	.46***	.74***	.25*	-.17	.05	.10	-.01	-.03	.15	.24	-.04	.75***	.61***

Note: Significance tests are 2-tailed. Perc. = Perceived

[a] Cronbach's alpha: perceived community improvement, α=.90; sustainability planning, α=.87. Cronbach's alpha was calculated on applicable measures using data from both Time 1 and Time 2.

*$p \leq .05$. **$p \leq .01$. ***$p \leq .001$.
